# Time-Dependent Trapping of Pollinators Driven by the Alignment of Floral Phenology with Insect Circadian Rhythms

**DOI:** 10.3389/fpls.2017.01119

**Published:** 2017-06-30

**Authors:** Jenny Y. Y. Lau, Xing Guo, Chun-Chiu Pang, Chin Cheung Tang, Daniel C. Thomas, Richard M. K. Saunders

**Affiliations:** ^1^School of Biological Sciences, The University of Hong KongHong Kong, China; ^2^Singapore Botanic GardensSingapore, Singapore

**Keywords:** Annonaceae, anthesis duration, floral phenology, insect circadian rhythms, pollinator trapping

## Abstract

Several evolutionary lineages in the early divergent angiosperm family Annonaceae possess flowers with a distinctive pollinator trapping mechanism, in which floral phenological events are very precisely timed in relation with pollinator activity patterns. This contrasts with previously described angiosperm pollinator traps, which predominantly function as pitfall traps. We assess the circadian rhythms of pollinators independently of their interactions with flowers, and correlate these data with detailed assessments of floral phenology. We reveal a close temporal alignment between patterns of pollinator activity and the floral phenology driving the trapping mechanism (termed ‘circadian trapping’ here). Non-trapping species with anthesis of standard duration (c. 48 h) cannot be pollinated effectively by pollinators with a morning-unimodal activity pattern; non-trapping species with abbreviated anthesis (23–27 h) face limitations in utilizing pollinators with a bimodal circadian activity; whereas species that trap pollinators (all with short anthesis) can utilize a broader range of potential pollinators, including those with both unimodal and bimodal circadian rhythms. In addition to broadening the range of potential pollinators based on their activity patterns, circadian trapping endows other selective advantages, including the possibility of an extended staminate phase to promote pollen deposition, and enhanced interfloral movement of pollinators. The relevance of the alignment of floral phenological changes with peaks in pollinator activity is furthermore evaluated for pitfall trap pollination systems.

## Introduction

Pollinator traps—defined as structures of a flower or inflorescence that prevent floral visitors from leaving until pollination has been achieved (Kugler, 1955)—have evolved independently in numerous angiosperm lineages, including basal angiosperms (Bolin et al., 2009; Oelschlägel et al., 2009), monocots (Vogel and Martens, 2000; Singer, 2002) and eudicots (Masinde, 2004; Heiduk et al., 2010). Most function as pitfall traps, in which floral visitors (generally insects) enter or fall into the flower or inflorescence and are trapped, often due to specialized trichomes and slippery surfaces that lack anchor points (Cammerloher, 1923, 1933; Daumann, 1971; Lack and Diaz, 1991; Vogel and Martens, 2000; Singer, 2002; Bolin et al., 2009; Oelschlägel et al., 2009; Heiduk et al., 2010), although a more complex trap mechanism operates in some sexually deceptive orchids (Hopper and Brown, 2006; Phillips et al., 2014). In order for pollination to be successful, however, it is essential that pollen-laden insects are able to escape; this can be achieved via specialized exit routes (Vogel and Martens, 2000; Singer, 2002; Nishizawa et al., 2005) or by structural modifications due to the wilting of organs or alteration of surface textures (Bolin et al., 2009; Oelschlägel et al., 2009).

**FIGURE 1:**
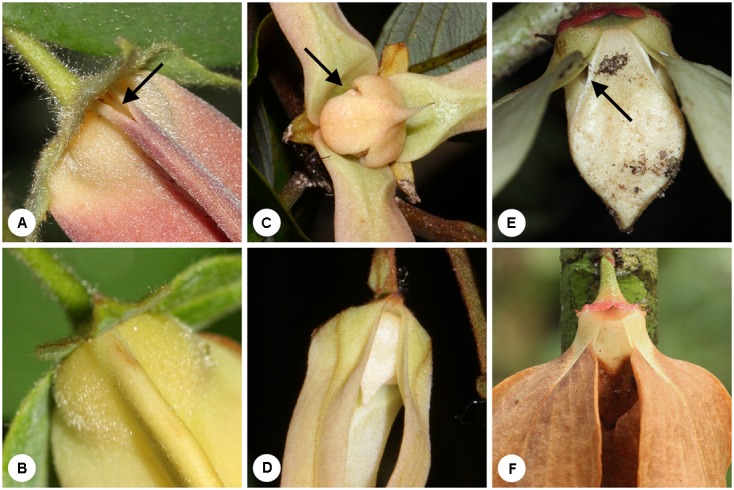
Pollinator trapping in Annonaceae flowers. **(A)** *Dasymaschalon trichophorum*, with open apertures (arrowed). **(B)** *Dasymaschalon trichophorum*, with apertures closed due to lateral growth of petals. **(C)** *Friesodielsia borneensis*, with open apertures (arrowed). **(D)** *Friesodielsia borneensis*, with apertures blocked due to movement of the outer petals. **(E)** *Goniothalamus tapisoides*, with open apertures (arrowed). **(F)** *Goniothalamus tapisoides*, with apertures blocked due to movement of the outer petals. (Photos **A,E,F**: J. Y. Y. Lau; **B**: C.-C. Pang; **C,D**: X. Guo).

Most extant early divergent angiosperms have hermaphroditic flowers that are protogynous, with many forming partially enclosed floral chambers (Thien et al., 2000; Saunders, 2010). The early divergent angiosperm family Annonaceae is dominated by species with a floral chamber, although the chamber generally does not function as a trap as pollinators are free to enter and leave throughout anthesis (Pang and Saunders, 2014). We have identified ‘true’ pollinator trapping in several disparate evolutionary lineages in the family, however, viz. *Goniothalamus* (Lau et al., 2016), the *Dasymaschalon-Friesodielsia* clade (Pang and Saunders, 2014; Guo, 2016), and possibly also *Artabotrys*; although additional field data are required, the trapping mechanisms are possibly synapomorphic for these clades and hence may represent key evolutionary innovations. The trapping mechanism in *Goniothalamus* flowers (**Figures 1E,F**) has been studied in detail (Lau et al., 2016). *Goniothalamus* flowers are trimerous, with morphologically distinct sepals, outer petals and apically connivent inner petals that form a floral chamber with three small basal apertures. The trap operates by the movement of alternately positioned outer petals so that the apertures are periodically blocked to prevent the exit of pollinators (Lau et al., 2016). Pollinator traps in *Friesodielsia* (**Figures 1C,D**) and *Artabotrys* are structurally similar to those of *Goniothalamus*, whereas those in *Dasymaschalon* (**Figures 1A,B**) differ since the flowers possess only one whorl of three petals. Although petals in *Dasymaschalon* are inferred to be homologous with the outer petals of other Annonaceae (Saunders, 2010), they are apically connivent and morphologically similar to the inner petals of the sister genus *Friesodielsia*; trapping operates by lateral petal expansion that closes the apertures between contiguous petals (Pang and Saunders, 2014).

The trap mechanism in these genera is therefore strikingly different from the pitfall traps reported in other angiosperms. Annonaceae pollinator traps are time-dependent (‘circadian traps’), with floral scent and/or the prospect of shelter and copulation tryst sites acting as baits to lure pollinators into the chamber. The chamber then closes due to the growth or movement of petals to prevent departure of the insects until pollen has been transferred to the stigma during the pistillate anthetic phase, and after pollen has been deposited on the insect during the subsequent staminate phase.

A correlation appears to exist in the Annonaceae between the occurrence of pollinator trapping and the duration of anthesis. Hermaphroditic Annonaceae flowers are protogynous and generally have anthesis over 36–54 h (Saunders, 2012), with a sexually non-functional interim phase separating the pistillate and staminate phases that effectively precludes intrafloral self-pollination (autogamy). The pollinators (primarily small beetles) are attracted to the flowers at the onset of the pistillate phase, with anthers subsequently dehiscing and then petals abscising at the end of anthesis. The end of anthesis generally coincides with the beginning of the pistillate phase in other flowers: pollen-laden beetles departing from post-staminate-phase flowers are therefore ideally timed for subsequent visits to pistillate-phase flowers, maximizing opportunities for successful pollination (Pang and Saunders, 2014). Pollinator-trapping Annonaceae flowers have been observed to have a shorter anthesis, however, viz. 23–27 h in *Dasymaschalon*, *Friesodielsia*, and *Goniothalamus* species (Pang and Saunders, 2014; Lau et al., 2016; this study). A similarly brief anthesis (c. 27 h) is reported for *Desmos chinensis* (Pang and Saunders, 2015), although this species does not trap pollinators. Annonaceae species with abbreviated anthesis often display pistillate/staminate-phase floral synchrony, in which pistillate-phase and staminate-phase flowers are not borne concurrently on an individual, hence minimizing opportunities for geitonogamy (Pang and Saunders, 2014, 2015; present study). We previously hypothesized that shortening of anthesis in species with this type of floral synchrony minimizes the proportion of non-flowering days and hence increases seedset (Lau et al., 2016).

Insect activity patterns are driven by endogenous periodicities and do not simply respond to environmental stimuli (Saunders, 2002). Although some insects possess a unimodal circadian rhythm with a single activity peak (Koskela, 1979), many possess a bimodal pattern with morning and evening peaks (Herrera, 1990; Armstrong and Marsh, 1997). We hypothesize that pollinator trapping in circadian trap flowers is likely to have evolved in response to the circadian rhythms of their pollinators. Although Annonaceae species are typically dependent on a single guild of pollinators (Saunders, 2012), the pollinators are not reciprocally dependent since they obtain nutrition from many different sources; the circadian traps are therefore likely to have resulted from evolutionary changes in plant phenology rather than changes in beetle circadian rhythms, and are not the result of co-evolution. The timing of anthesis and floral-reward production within each day are likely shaped by the activity peaks of pollinators in order to maximize pollination efficiency (Stebbins, 1970; Armbruster and McCormick, 1990; Herrera, 1990; Jagadish et al., 2002; Kulloli et al., 2011; Prieto-Benítez et al., 2016). Significantly, however, studies of the relationship between the circadian rhythms of pollinators and floral phenology are generally lacking: although previous assessments of pollinator activity have relied on monitoring of floral visits, it has been suggested that in order to study the evolution of floral changes in response to pollinator activity it is more appropriate to assess pollinator activities independently of their interaction with flowers (Armbruster and McCormick, 1990).

Our primary objective is therefore to investigate whether changes in the timing of anthesis and the occurrence of pollinator trapping in Annonaceae flowers are associated with different pollinator circadian rhythms: we hypothesize that the evolutionary alignment of floral phenology with the circadian rhythm of the primary pollinator can enhance the efficacy of pollination. In order to test this, we monitored the activity patterns of beetle pollinators retrieved from Annonaceae flowers with contrasting phenologies and pollinator trapping abilities: (a) species with anthesis of standard duration that cannot trap pollinators (*Polyalthia suberosa*); (b) species with abbreviated anthesis that similarly cannot trap pollinators (*Desmos chinensis*); and (c) species with abbreviated anthesis that trap pollinators (*Dasymaschalon trichophorum*, *Friesodielsia borneensis*, and *Goniothalamus tapisoides*).

## Materials and Methods

### Study System and Sampling of Species

We selected five Annonaceae species that exhibit differing durations of anthesis and presence/absence of pollinator trapping, viz.: (a) *Polyalthia suberosa* (Roxb.) Thwaites: anthesis over c. 48 h, pollinated by curculionid beetles (this study), lacking any trapping mechanism; (b) *Desmos chinensis* Lour.: anthesis over c. 27 h, pollinated by nitidulid beetles (Pang and Saunders, 2015), lacking trapping; (c) *Dasymaschalon trichophorum* Merr.: anthesis over c. 26 h, pollinated by curculionid beetles (Pang and Saunders, 2014), with pollinator trapping; (d) *Friesodielsia borneensis* (Miq.) Steenis: anthesis over c. 26 h, pollinated by a combination of curculionid, cf. *Carpophilus* (Nitidulidae) and staphylinid beetles (this study), with pollinator trapping; and (e) *Goniothalamus tapisoides* Mat-Salleh: anthesis over c. 23 h, pollinated by curculionid and nitidulid beetles (Lau et al., 2016), with pollinator trapping. The study localities, beetle pollinator identities and sample sizes are indicated in **Table 1**.

**Table 1:** Field locations and sampling size of the pollinators of selected Annonaceae species.

	*Polyalthia suberosa*	*Desmos chinensis*	*Dasymaschalon trichophorum*	*Friesodielsia borneensis*	*Goniothalamus tapisoides*
Field site	Daoyin village, Yinggeling Nature Reserve, Hainan; 18°59′03′′N, 109°19′45′′E	Mui Tsz Lam Road, Ma On Shan, Hong Kong; 22°24′00.24′′N, 114°13′39.96′′E	Daoyin village, Yinggeling Nature Reserve, Hainan; 18°59′03′′N, 109°19′45′′E	Nee Soon, Singapore; 01°23′52.80′′N, 103°48′32.43′′E	Rampayoh, Brunei Darussalam; 04°22′03.3′′N, 114°27′37.9′′E
Sampling period	May 2015 (10 days)	June 2014 (13 days)	May 2015 (20 days)	June 2015 (20 days)	May 2014 (21 days)
Beetle species and sample sizes	Curculionidae sp. (*n* = 8)	*Amystrops* sp. (Nitidulidae) (*n* = 48)	*Endaenidius* cf. *polyalthiae* (Curculionidae) (*n* = 27)	(a) cf. *Carpophilus* sp. (Nitidulidae) (*n* = 7);	(a) *Endaeus* sp. 1 (Curculionidae) (*n* = 20);
				(b) Curculionidae sp. (*n* = 3);	(b) *Endaeus* sp. 2 (Curculionidae) (*n* = 22);
				(c) Staphylinidae sp. (*n* = 6)	
					(c) *Endaeus* sp. 3 (Curculionidae) (*n* = 13);
					(d) cf. *Carpophilus* sp. (Nitidulidae) (*n* = 11)

### Assessment of Flower-Level Phenology and Pollination Ecology

Phenological and pollination ecology data for *Desmos chinensis* and *Goniothalamus tapisoides* are already published (Pang and Saunders, 2015; Lau et al., 2016), with new data presented here for *Dasymaschalon trichophorum*, *Friesodielsia borneensis*, and *Polyalthia suberosa*. Populations were monitored using localities detailed in **Table 1**, with phenological data for *P. suberosa* supplemented using cultivated individuals in the South China Botanic Garden.

Flower-level phenology for the previously unstudied species was assessed following extensive surveys at field sites in Hainan and Singapore (permits NP/RP14-043 and NP/RP15-010 from the National Parks Board, Singapore) (**Table 1**): *F. borneensis* was assessed using 125 flower buds from 12 individuals over 21 days in June–July 2014; *D. trichophorum* using 80 flower buds from 10 individuals over 20 days in May 2010 and seven days in May 2013; and *P. suberosa* using 30 flower buds from five individuals over 10 days in May 2015 and eight days in June 2016. Flower buds were tagged and monitored daily until anthesis, with subsequent observations at 2-h intervals. Stigmatic receptivity was determined by immersing stigmas in 3% hydrogen peroxide (H_2_O_2_) to assess peroxidase enzyme activity (Galen and Plowright, 1987). Concatenating flower-level phenological data from different individuals, including the occurrence of pistillate- and staminate-phase flowers, enabled an assessment of intra- and inter-individual floral synchrony.

Floral visitors to each species were assessed throughout the study period, with activities recorded at 2-h intervals. Samples were caught with an entomological net, immobilized with chloroform, and dried using silica gel for subsequent assessment of the presence of pollen grains and taxonomic identification. Determination of effective pollination was based on the following criteria: relative visitation rates; coincidence of visits with the floral reproductive phases; attachment of pollen grains; and evidence of interfloral movement. Since hermaphroditic Annonaceae flowers are protogynous, the latter criterion was indicated by the presence of pollen-laden beetles in pistillate-phase flowers.

### Assessment of Pollinator Circadian Rhythms

The daily activity patterns of beetles were monitored using a Drosophila Activity Monitor (DAM; Trikinetics, Waltham, MA, United States). Beetle pollinators were collected from within the floral chambers of anthetic flowers over extended periods of fieldwork at sites in Brunei Durassalam (permit 53/JPH/BOT/02 PT.3 issued by Forestry Department, Ministry of Industry and Primary Resources), Hainan, Hong Kong and Singapore (permits NP/RP14-043 and NP/RP15-010 from the National Parks Board, Singapore) (**Table 1**). The beetles were inserted into the vials immediately and not provided with water or nutrients during the monitoring period. Their activity patterns (assessed as interruptions of the infrared beams that bisect the glass vials) were monitored continuously at 5-min intervals under ambient temperature and natural photoperiod for at least three days. Data gathered on the first day was discarded to ensure acclimation of beetles. DAM Filescan102X (Trikinetics, Waltham, Massachusetts, USA) was used to aggregate raw data into 15-min intervals for each individual. The activity levels of the replicates (**Table 1**) were averaged to generate a daily activity pattern graph for each species.

### Identification of Insect Pollinators

The taxonomic identities of beetle pollinators follow previous studies (Pang and Saunders, 2015; Lau et al., 2016) where available, with the pollinators of other species identified by entomologists from Natural History Museum, London (*Dasymaschalon trichophorum*) or by DNA barcoding (*Friesodielsia borneensis* and *Polyalthia suberosa*). Silica-gel dried insect samples were dissected and immersed in 200 μl buffer AE overnight. Total genomic DNA was extracted using DNeasy Blood&Tissue Kit (Qiagen), following the manufacturer’s protocol. Fragments from the mitochondrial cytochrome *c* oxidase subunit I gene (COI) were amplified using primers LCO1490: 5′-GGTCAACAAATCATAAAGATATTGG-3′ and HCO2198: 5′-TAAACTTCAGGGTGACCAAAAAATCA-3′ (Folmer et al., 1994).

Amplification was performed using the GoTag Flexi DNA Polymerase package (Promega, Madison, WI, USA) with the preparation of 20 μl reactions containing 10.04 μl ddH_2_O, 4 μl 5× reaction buffer, 2.4 μl MgCl_2_ (25 mM), 0.4 μl dNTPs (10 mM each), 0.6 μl of each forward and reverse primer (10 μM), 1 μl bovine serum albumin (BSA, 10 mg/ml), 0.16 μl of Flexitaq DNA polymerase (Promega, Madison, WI, United States), and 0.8 μl of DNA template. The PCR samples were then processed in a GeneAmp PCR System 2700 (Applied Biosystems, Foster City, CA, United States) under the following conditions: initial denaturation at 94°C for 5 min, 30 cycles of primer denaturation at 94°C for 1 min; annealing at 55°C for 1 min; and extension at 72°C for 1 min, with a final extension at 72°C for 10 min. PCR products were then purified using QIAquick PCR purification kit (Qiagen Inc., Valencia, CA, United States) according to the manufacturer’s instructions. Amplification products were sent for commercial sequencing to the Beijing Genomics Institute (BGI, Hong Kong, China). Sequencing products for bidirectional sequencing were generated using the same primers as above and the BigDye Terminator Cycle Sequencing Kit (Applied Biosystems, Foster City, CA, United States), and run on an Applied Biosystems 3730 XL DNA Analyser. BOLD Identification Engine^1^ and BLAST^2^ searches were used for nucleotide sequence comparison against major databases.

## Results

### Floral Phenology and Pollination Ecology of *Polyalthia suberosa*

*Polyalthia suberosa* is protogynous, with anthesis over c. 48 h. The following four distinct stages are evident:

#### Stage I: Pre-receptive Phase

The outer petals are light green and reflexed outwards. The inner petals are initially greenish-yellow and mutually separated, but subsequently become a more intense yellow and close to form a loose chamber around the reproductive organs prior to the onset of the pistillate phase.

#### Stage II: Pistillate Phase (c. 15 h Duration)

The inner petals are loosely contiguous, forming an apical aperture with three basal apertures. The stigmas are receptive from c. 18:00 h, as evidenced by the formation of stigmatic exudate, and a sweet fruity odor is emitted.

#### Stage III: Interim Phase (c. 7 h Duration)

The apertures in the floral chamber remain open. The stigmatic exudate begins to dry around 09:00 h on the second day, reflecting a decrease in stigmatic receptivity. The carpels turn pink, the stamens turn brown, and the fruity scent diminishes.

#### Stage IV: Staminate Phase (c. 14 h Duration)

The apertures in the floral chamber remain open. Anther dehiscence starts around 16:00 h on Day 2 and is associated with the emission of a fruity scent. The petals and stamens abscise around 18:00 h, indicating the end of the staminate phase.

Ten curculionid individuals, some of which were bearing pollen grains of *P. suberosa*, were retrieved from pistillate-phase flowers (Daoyin village, Yinggeling Nature Reserve, Hainan, 2015), providing unequivocal evidence of interfloral pollinator movement. Although this curculionid species is undoubtedly a pollinator of *P. suberosa*, some *Carpophilus* cf. *marginellus* individuals were also observed to visit the flowers. As basal and apical apertures remain open throughout anthesis, the pollinators are free to enter and exit the flowers at any stage.

### Floral Phenology and Pollination Ecology of *Dasymaschalon trichophorum*

*Dasymaschalon trichophorum* is protogynous, with anthesis over c. 25 h. The following four distinct stages are evident:

#### Stage I: Pre-receptive Phase

The flower buds remain green until late in bud development, when most parts of the petals (except the base) become red. The apertures at the base of pollination chamber remain closed.

#### Stage II: Pistillate Phase (c. 7 h Duration)

The floral chamber apertures are open throughout this stage. The stigmas become receptive around 04:00 h with the formation of stigmatic exudate. No floral scent is detectable by human perception.

#### Stage III: Interim Phase (c. 4 h Duration)

The floral chamber apertures remain open. Stigmatic receptivity diminishes with drying of stigmatic exudate from c. 11:00 h. No floral scent is apparent.

#### Stage IV: Staminate Phase (c. 15 h Duration)

The floral chamber apertures close around 16:30 h due to the lateral expansion of petals, which begins from the adaxial surface of the petal and extends outwards. Anther dehiscence occurs around 15:00 h, with all stamens and petals abscising by the end of this stage.

In total, 31 and 27 individuals of *Endaenidius* cf. *polyalthiae* (Curculionidae) beetles were collected from flowers during fieldwork in Daoyin village, Yinggeling Nature Reserve, Hainan in May 2010 and 2015, respectively. Pollen grains were found on beetles that were retrieved from pistillate-phase flowers, providing unequivocal evidence of inter-floral movement of beetles. As the basal apertures close around 16:30 h, near the onset of the staminate phase, pollinators are trapped inside floral chamber from that point on and are only released at the end of staminate phase with the abscission of the corolla.

### Floral Phenology and Pollination Ecology of *Friesodielsia borneensis*

*Friesodielsia borneensis* is protogynous, with anthesis over c. 26 h. The following four distinct stages are evident:

#### Stage I: Pre-receptive Phase

The outer petals are initially apically connivent, but begin to separate from the base to the apex, ultimately exposing the inner petals the day before the onset of the pistillate phase. The outer petals subsequently reflex outwards, exposing the three basal apertures in the floral chamber towards the beginning of the pistillate phase.

#### Stage II: Pistillate Phase (c. 6 h Duration)

The three basal apertures in the floral chamber are initially fully open. The outer petals subsequently return to their original position from c. 08:00 h, however, so that they are closely appressed against the inner petal dome and hence block the three apertures in the floral chamber. The stigmas are receptive from c. 05:00 h with evidence of stigmatic exudate formation. A fruity floral scent is emitted.

#### Stage III: Interim Phase (c. 11 h Duration)

The three floral chamber apertures remain blocked by the outer petals. The stigmas become dry and are no longer receptive from c. 11:00 h. The fruity floral scent dissipates.

#### Stage IV: Staminate Phase (c. 9 h Duration)

The floral chamber apertures remain closed. The anthers dehisce from c. 22:00 h and no floral scent is apparent. The petals and stamens abscise at the end of the staminate phase.

Sexual maturation of flowers was monitored on six individuals over a 10-day period, and was generally observed to occur every second day, separated by one flowerless day, with only four out of 79 flowers (5.1%) observed to flower on consecutive days (Supplementary Table S1). This is clear evidence of pistillate/staminate-phase floral synchrony: in this case, pistillate-phase flowers are unlikely to be pollinated by pollen from staminate-phase flowers borne on the same individual, and hence the possibility of geitonogamy is reduced.

Three small beetle species were observed to visit *F. borneensis* flowers and to be trapped inside mature flowers during field observations in Nee Soon, Singapore, in June to July 2014 and 2015, viz. a curculionid species (eight individuals), a cf. *Carpophilus* species (32 individuals) and a staphylinid species (six individuals). Pollen grains were found attached to the curculionid, cf. *Carpophilus* and staphylinid beetles retrieved from pistillate-phase flowers. Beetle pollinators are trapped inside the pollination chamber from 08:00 h due to the closure of basal apertures in the floral chamber, and are only released after the abscission of petals at the end of staminate phase, c. 07:00 h the following day.

### Assessment of Pollinator Circadian Rhythms

**FIGURE 2:**
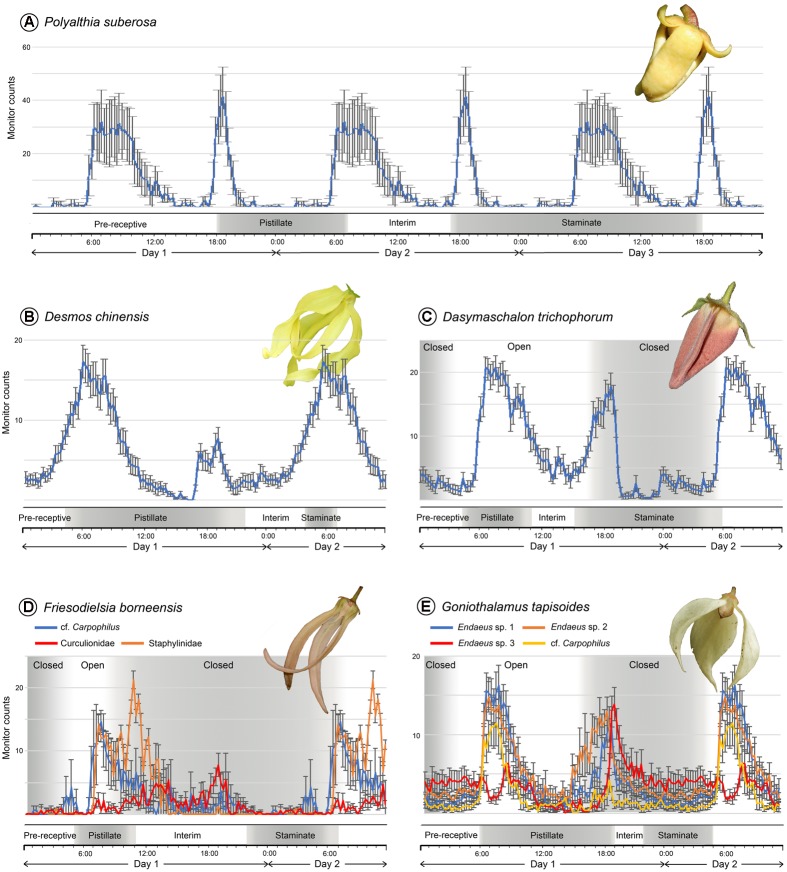
Floral phenology and daily activity patterns of beetle pollinators of five Annonaceae species, obtained using a *Drosophila* Activity Monitor. **(A)** Pollinator of *Polyalthia suberosa*: Curculionidae sp. (*n* = 8). **(B)** Pollinator of *Desmos chinensis*: *Amystrops* sp., Curculionidae (*n* = 48); phenology bar redrawn from Pang and Saunders (2015). **(C)** Pollinator of *Dasymaschalon trichophorum*: *Endaenidius cf. polyalthiae*, Curculionidae (*n* = 27); phenology bar redrawn from Pang and Saunders (2014). **(D)** Pollinators of *Friesodielsia borneensis*: cf. *Carpophilus* sp., Nitidulidae (*n* = 7); Staphylinidae sp. (*n* = 6); Curculionidae sp. (*n* = 3). **(E)** Pollinators of *Goniothalamus tapisoides*: *Endaeus* sp. 1, Curculionidae (*n* = 20); *Endaeus* sp. 2, Curculionidae (*n* = 22); *Endaeus* sp. 3, Curculionidae (*n* = 13); *cf. Carpophilus* sp., Nitidulidae (*n* = 11); phenology bar redrawn from Lau et al. (2016). The activity patterns were reconstructed over a 24-h period and the data repeated in the diagram over subsequent days. Error bars show standard error.

The small curculionid beetles that are inferred to be effective pollinators of *Polyalthia suberosa* show a clear bimodal circadian rhythm (**Figure 2A**): they were most active in the morning, around 06:00–09:00 h (average 30 counts per 15 min), although there is another activity peak in the evening, around 19:00 h (42 counts per 15 min) of a shorter duration.

The *Amystrops* beetle species (Nitidulidae), which pollinates *Desmos chinensis*, displays a weakly bimodal circadian rhythm (**Figure 2B**): the beetles were most active in the morning, around 06:00–08:00 h (average 14–17 counts per 15 min), although a lower intensity activity peak was recorded during the evening, from 17:30–19:00 h (4–5 counts per 15 min).

Only one beetle species, *Endaenidius* cf. *polyalthiae* (Curculionidae), has been recorded as an effective pollinator of *Dasymaschalon trichophorum*. This species shows a very clear bimodal circadian rhythm (**Figure 2C**): they were very active in the early morning, from 06:00 to 08:00 h (average 21 counts per 15 min), and again during the evening, from 18:00 to 19:00 h (18 counts per 15 min).

Three beetle species have been identified as pollinators of *Friesodielsia borneensis*, including two that were most active in the morning: the cf. *Carpophilus* (Nitidulidae) species was active from 07:00 to 08:00 h (average 15 counts per 15 min); and the staphylinid species showed peak activity around 10:00–11:00 h (22 counts per 15 min) (**Figure 2D**). The curculionid species was active from noon until evening, however, peaking around 1300–1400 h (seven counts per 15 min), and again around 19:00 h (10 counts per 15 min). Two species therefore show a morning circadian rhythm, whereas the third is weakly bimodal.

Four beetle species are confirmed as effective pollinators of *Goniothalamus tapisoides*, viz.: three *Endaeus* species (Curculionidae) and a species resembling *Carpophilus* (Nitidulidae). Among these, *Endaeus* spp. 1 and 2 show very similar bimodal circadian rhythms with high counts in the morning, peaking at 07:00 h (average 15 counts per 15 min), and in the evening, peaking at 19:00 h (11 counts per 15 min; **Figure 2E**). *Endaeus* sp. 3, in contrast, shows an activity peak in the evening, around 19:00 h (14 counts per 15 min), but lacks a significant peak in the morning (**Figure 2E**). The fourth pollinator, cf. *Carpophilus* sp., has the reverse activity pattern, peaking in the morning around 07:00 h (12 counts per 15 min) and lacking any significant evening peak (**Figure 2E**). *Endaeus* spp. 1 and 2 therefore possess bimodal circadian rhythms, whereas *Endaeus* sp. 3 has an evening unimodal activity pattern, and cf. *Carpophilus* sp. has a morning unimodal pattern.

## Discussion

### Floral Phenology and Pollinator Circadian Rhythms in the Absence of Pollinator Trapping

*Polyalthia suberosa* was selected as an exemplar small-beetle pollinated Annonaceae species exhibiting a standard anthesis duration (c. 48 h). The curculionid beetle pollinators are shown to have a bimodal circadian rhythm (**Figure 2A**). The onset of the pistillate anthetic phase (17:00 h on Day 1) is associated with the emission of floral scents that attract beetles during their peak activity in the evening (from 18:00 h); pollen-laden beetles that had previously visited staminate-phase flowers are therefore able to transfer pollen to receptive stigmas. The beetles subsequently remain relatively immobile in the flower until their next activity peak the following morning (06:00–09:00 h of Day 2); this coincides with the end of the pistillate phase, at which point the dissipation of floral scent encourages pollinator departure. A new cohort of beetles is attracted during their next activity peak (from 18:00 h), which coincides with the onset of the staminate phase when the flowers again emit a scent. Beetles subsequently leave the flower laden with pollen either in the morning or evening of Day 3. Although our study is the first to investigate the circadian rhythms of beetle pollinators, the anthesis duration and pollination ecology pattern described above for *P. suberosa* appears to be typical for most beetle-pollinated Annonaceae species (Ratnayake et al., 2006, 2007; Saunders, 2010; Weerasooriya and Saunders, 2010). Beetles that exhibit a morning-unimodal circadian rhythm are unlikely to be effective pollinators due to poor alignment of peak activity with the functional sexual phases of the flower.

*Desmos chinensis* is also pollinated by small beetles and similarly lacks a pollinator trapping mechanism. It differs from *P. suberosa*, however, in having an abbreviated anthesis of only c. 27 h (Pang and Saunders, 2015): it has a lengthy pistillate phase (c. 18 h), followed by a non-sexual interim phase (c. 6 h) and then a comparatively brief staminate phase (c. 3 h). The nitidulid beetle pollinators show a weakly bimodal circadian rhythm (**Figure 2B**): they are very active during the early pistillate phase (from 04:00 h on Day 1) and are attracted by the scent emitted by the flowers. Beetle movement persists through the early pistillate phase, with a weak evening peak in activity (17:30–20:00 h of Day 1) occurring before the end of the pistillate phase, providing an extended opportunity for pollen transfer to the stigmas. The next major activity peak (06:00 h of Day 2) coincides with the onset of the staminate phase, thereby maximizing opportunities for pollen deposition on the beetles prior to their departure following petal abscission at the end of anthesis. Pollination in *D. chinensis* is therefore largely achieved by beetles that display a morning-unimodal circadian rhythm: bimodally active beetles would be less effective pollinators as they would likely depart from pistillate-phase flowers in the evening at a time when complementary staminate-phase flowers are unavailable. The evolutionary disadvantages arising from this limitation to the diversity of potential effective pollinators based on their circadian rhythms would be offset by the increased seedset due to more rapid turnover of flowers.

### Floral Phenology and Pollinator Circadian Rhythms in Association with Pollinator Trapping

The abbreviated anthesis evident in *Desmos chinensis* (c. 27 h duration: Pang and Saunders, 2015) is also apparent in *Dasymaschalon trichophorum* (c. 26 h duration: Pang and Saunders, 2014) and *Friesodielsia borneensis* (c. 26 h duration: this study). These three genera consistently form a well-supported clade in recent molecular phylogenies (Guo et al., 2017), with *Desmos* sister to the *Dasymaschalon-Friesodielsia* clade. Abbreviated anthesis is therefore possibly synapomorphic for the entire clade and can be inferred to have evolved prior to pollinator trapping in the *Dasymaschalon-Friesodielsia* lineage. The structural differences in floral morphology between *Dasymaschalon* and *Friesodielsia* suggest that pollinator trapping may have arisen by evolutionary convergence and hence not be homologous. Alternatively, the morphological similarities between the petals in *Dasymaschalon* (inferred as homologues of outer petals: Saunders, 2010) and the inner petals in *Friesodielsia* might be explained by a disruption of the homeotic genetic control of organ identity during floral development (Saunders, 2010).

*Dasymaschalon trichophorum* is pollinated by curculionid beetles that show a bimodal circadian rhythm. The onset of the pistillate phase (from 04:30 h on Day 1) is associated with the emission of floral scents (Pang and Saunders, 2014) that attract beetles as they become active in the morning (06:00–08:00 h; **Figure 2C**). The beetles are able to enter the floral chamber via small apertures between the petals (**Figure 1A**), but are trapped by the time the beetles become active again in the evening (18:00–19:00 h), prolonging the period for pollen deposition. The closure of the aperture begins from the adaxial petal surface and extends outwards, and hence beetles are prevented from leaving the floral chamber before the external margin of the aperture closes: this explains the apparent discrepancies in the timing of aperture closure reported here (c. 15:30 h; **Figure 1E**) and previously (c. 22:00 h: Pang and Saunders, 2014). The subsequent activity peak on the morning of Day 2 coincides with the extended staminate phase (c. 15 h), further enhancing opportunities for pollen deposition as the beetles move around within the enclosed floral chamber. Since the floral scents likely mimic food or sexual pheromones (Pang and Saunders, 2014), trapping beetles until other flowers enter their pistillate phase would possibly minimize pollinator loss.

The pollination ecology of *Friesodielsia borneensis* resembles that of *D. trichophorum*, although the former is pollinated by a greater diversity of beetle species. The cf. *Carpophilus* and staphylinid beetles exhibit unimodal circadian rhythms, with morning activity peaks (07:00–08:00 and 10:00–11:00 h, respectively) that correspond with the pistillate anthetic phase (05:00–11:00 h; **Figure 2D**). The floral chamber closes around 08:00 h, prior to the end of the pistillate phase and the dissipation of floral scents and pollinator food reward. The beetles are therefore trapped in the flower until the end of the staminate phase when the petals abscise; this coincides with the morning peak in the activity of the cf. *Carpophilus* and staphylinid beetles on Day 2, promoting the departure of pollen-laden beetles. The curculionid species shows a weakly bimodal circadian rhythm, although relatively few individuals were observed during fieldwork due to limitations in the availability of anthetic flowers.

The evolutionary importance of circadian pollinator trapping in the Annonaceae is apparent from its independent evolutionary origin in the phylogenetically distant genus *Goniothalamus*. A diverse range of beetle species were observed to pollinate *Goniothalamus tapisoides*, including two (*Endaeus* spp. 1 and 2) that show a bimodal circadian rhythm, one (*Endaeus* sp. 3) that has an evening unimodal pattern, and one (cf. *Carpophilus*) with a morning unimodal pattern (**Figure 2E**). The floral chamber is open as the pistillate phase begins in the early morning (from 06:00 h). Three of the pollinator species (*Endaeus* spp. 1 and 2, and cf. *Carpophilus*) are active at this time and so are likely attracted to the flowers in search of food (presumably stigmatic exudate); those that had previously visited staminate-phase flowers would therefore be able to transfer pollen to the receptive stigmas. The activity levels of these beetles gradually diminish during the day, but peak again around 18:00–19:00 h; since this coincides with the end of the pistillate phase (and hence loss of the floral scent and food source) the beetles may attempt to leave the flowers. The floral chamber apertures are blocked by the movement of the outer petals from 15:00 h, however, and so the beetles are trapped inside the chamber throughout the remainder of anthesis. Pollen released during the staminate phase is therefore likely to be deposited on the pollinators since the beetles would be actively moving around the enclosed chamber.

*Endaeus* spp. 1 and 2 and the cf. *Carpophilus* sp. are all active in the early pistillate phase prior to the closing of the floral trap, and are therefore likely to be effective pollinators. *Endaeus* sp. 3, however, shows an evening unimodal activity pattern and is therefore unlikely to be as effective since few individuals would be active at the start of the pistillate phase.

### Evolutionary Significance

Our results show a clear relationship between the duration of anthesis, the occurrence of a pollinator trapping mechanism, and the circadian rhythm of the pollinating beetles. Non-trapping flowers with anthesis of standard duration (e.g., *Polyalthia suberosa*) cannot be pollinated effectively by pollinators with a morning-unimodal activity pattern due to poor alignment of peak activity with the functional sexual phases of the flower. Non-trapping species with abbreviated anthesis (e.g., *Desmos chinensis*) face limitations in utilizing pollinators with a bimodal circadian activity, with beetles likely to depart from pistillate-phase flowers in the evening at a time when complementary staminate-phase flowers are unavailable, and hence less effective as pollinators. Species that trap pollinators (all with short anthesis) can utilize a broader range of potential pollinators, however, including those with both unimodal and bimodal circadian rhythms: in addition to identifying bimodally active pollinators for all three trapping species studied, we also highlight morning-unimodal pollinators of *Friesodielsia borneensis* (Nitidulidae and Staphylinidae) and *Goniothalamus tapisoides* (Nitidulidae), and evening-unimodal pollinators of *G. tapisoides* (Curculionidae).

A clear relationship furthermore exists in the Annonaceae between anthetic duration and the occurrence of pistillate/staminate-phase floral synchrony, in which pistillate-phase and staminate-phase flowers do not co-occur on an individual. Although *Maasia* (Rogstad, 1994, as ‘*Polyalthia*’) exhibits floral synchrony and has flowers that are anthetic over two days, most Annonaceae species that have been demonstrated to show floral synchrony have abbreviated anthesis, viz. species of *Annona* (Murray and Johnson, 1987, as ‘*Rollinia*’; Lora et al., 2011; Kishore et al., 2012), *Dasymaschalon* (Pang and Saunders, 2014), *Desmos* (Pang and Saunders, 2015), and *Friesodielsia* (this study). It should be noted, however, that studies of floral synchrony in the family are limited, and data are not available for many species known to have abbreviated anthesis.

The Annonaceae lack a genetic self-incompatibility mechanism but have several mechanisms that promote xenogamy, including protogyny, which precludes autogamy, and pistillate/staminate-phase floral synchrony, which minimizes opportunities for geitonogamy (Pang and Saunders, 2014). The functioning of pistillate/staminate-phase floral synchrony requires non-flowering days to ensure temporal separation of pistillate- and staminate-phase flowers: the negative impact of this on reproductive capacity is offset by shortening the duration of anthesis, and this presumably explains the close association between the two phenomena. There is no evidence of floral synchrony in *Goniothalamus*, however, despite exhibiting abbreviated anthesis (Lau et al., 2016): although geitonogamy is therefore possible, it is likely minimized by the limited number of flowers that reach maturity on consecutive days.

The evolution of abbreviated anthesis is furthermore closely associated with the evolution of pollinator trapping: it appears that most genera with abbreviated anthesis have pollinator trapping mechanisms, with the exception of some *Annona* (Kishore et al., 2012), *Desmos*, and *Duguetia* (Silberbauer-Gottsberger et al., 2003) species (although we only have circumstantial evidence for trapping in *Artabotrys*). This implies that pollinator trapping endows a major selective advantage: this may be the utilization of a broader range of beetle pollinators, including those with bimodal circadian rhythms. As beetles with bimodal patterns have activity peaks in the early morning and evening, a trap is necessary to retain them inside the flower in order to achieve optimal pollen loading. The phylogenetic position of *Desmos* (sister to the *Dasymaschalon*-*Friesodielsia* clade: Guo et al., 2017) and its lack of pollinator trapping suggests that abbreviated anthesis may have evolved in the clade prior to pollinator trapping.

The floral phenological patterns elucidated here for five Annonaceae species suggest that the timing of key events—the end of the staminate phase, the onset of the pistillate phase in other flowers, and the peak activity periods of the beetle pollinators—all coincide at approximately the same time in the early morning (**Figure 2**). The circadian rhythms of the beetles enable efficient pollination by promoting interfloral movement from staminate-phase flowers directly to flowers entering their pistillate phase. Although this is also true of *Polyalthia suberosa*, which has a typical anthesis duration (**Figure 2A**), it is possible for beetles to leave flowers of this species at the end of the pistillate phase (09:00 h) and move to another flower that is entering its pistillate phase, thereby reducing the efficiency of pollen transfer.

The study species with pollinator trapping have an extended staminate phase (*Dasymaschalon trichophorum*: c. 17 h, **Figure 2C**; *Friesodielsia borneensis*: c. 9 h, **Figure 2D**; *Goniothalamus tapisoides*: c. 7 h, **Figure 2E**) due to the early dehiscence of anthers, and as a consequence pollen load on the pollinators is potentially increased. As the trapping mechanism delays pollinator departure until the onset of the pistillate phase in other flowers, pollen loading can be further optimized. *Desmos chinensis*, in contrast, has a brief staminate phase (c. 3 h, **Figure 2B**) restricted to the early morning; since the flowers lack a pollinator trap, the beetles can leave the flowers at any time and premature anther dehiscence would merely result in pollen-laden beetles being unable to locate corresponding pistillate-phase flowers, leading to pollen wastage.

### Comparison of Circadian and Pitfall Pollinator Traps

The novel circadian trap mechanism described here for the Annonaceae is strikingly different from pitfall traps in other angiosperms (Vogel and Martens, 2000; Masinde, 2004; Bolin et al., 2009; Oelschlägel et al., 2009): pitfall traps allow continuous entry of insects during anthesis and merely prevent their early departure (Oelschlägel et al., 2009), whereas in Annonaceae closure of the chamber prevents pollinator entry and departure and is synchronized with the circadian rhythms of the pollinators. The blocking of the floral chamber apertures is shown to be closely aligned with the pollinators’ daily activity peaks. Since pitfall pollinator traps never completely close, it is less likely that the circadian rhythm of the pollinator will drive pollinator selection in the same way as in the Annonaceae.

Pollinators in Annonaceae circadian trap flowers can only leave when the petals abscise at the end of anthesis, within a very brief period (Pang and Saunders, 2014; Guo, 2016; Lau et al., 2016) that is closely aligned with the beetles’ peak activity (**Figures 2C–E**). Pollinators are generally released from pitfall traps over a considerably longer period, however: 3 days in *Hydnora africana* (Hydnoraceae/Aristolochiaceae; Bolin et al., 2009), 6 days in *Arisaema tortuosum* (Araceae; Vogel and Martens, 2000), and 3 days in *Trigonidium obtusum* (Orchidaceae; Singer, 2002). Some pitfall traps in *Aristolochia* flowers (Aristolochiaceae) differ, however, with trapped insects released within one day (Razzak et al., 1992; Oelschlägel et al., 2009) or 50–53 h (Murugan et al., 2006) after the stigmas cease receptivity. It is therefore possible that the timing of pollinator release from *Aristolochia* pitfall traps might also be aligned with the peak activity of the pollinators: the results of the present study of circadian pollinator traps in the Annonaceae are therefore possibly applicable to some pitfall traps.

## Author Contributions

JL, XG, C-CP, and RS conceived the study; JL, XG, and C-CP undertook experiments (with assistance from CT, and DT); JL analyzed the data; JL and RS wrote the initial manuscript; and all authors reviewed and edited the final submission.

## Conflict of Interest Statement

The authors declare that the research was conducted in the absence of any commercial or financial relationships that could be construed as a potential conflict of interest.

## Footnotes

1 http://www.boldsystems.org

2 https://blast.ncbi.nlm.nih.gov/Blast.cgi
