# Supplementary material for: Time-Dependent Trapping of Pollinators Driven by the Alignment of Floral Phenology with Insect Circadian Rhythms
Source: Front Plant Sci. 2017 Jun 30;8:1119. doi: 10.3389/fpls.2017.01119 (PMC5491900; doi:10.3389/fpls.2017.01119)
Supplement: Supplementary file 1 [file Table_1.DOCX]

**Table S1. Number of pistillate-phase (♀) and staminate-phase (♂) flowers borne on six individuals of *Friesodielsia borneensis*, monitored over 10 consecutive days.** Co-occurrence of pistillate- and staminate-phase flowers are shown in bold.

|  | **Individual** | | | | | |
| --- | --- | --- | --- | --- | --- | --- |
|  | **1** | **2** | **3** | **4** | **5** | **6** |
| **Day/Month** | **♀, ♂** | **♀, ♂** | **♀, ♂** | **♀, ♂** | **♀, ♂** | **♀, ♂** |
| 21/6 | 0, ? | 8, ? | 0, ? | 0, ? | 0, ? | 0, ? |
| 22/6 | 0, 0 | 0, 8 | 1, 0 | 3, 0 | 1, 0 | 4, 0 |
| 23/6 | 0, 0 | 6, 0 | **1**, **1** | 1, 3 | 0, 1 | 0, 4 |
| 24/6 | 0, 0 | **1**, **6** | 0, 1 | **1**, **1** | 3, 0 | 4, 0 |
| 25/6 | 0, 0 | 0, 1 | 0, 0 | **1**, **1** | 0, 3 | 0, 4 |
| 26/6 | 0, 0 | 9, 0 | 3, 0 | 0, 1 | 0, 0 | 2, 0 |
| 27/6 | 0, 0 | 0, 9 | 0, 3 | 2, 0 | 0, 0 | 0, 2 |
| 28/6 | 1, 0 | 14, 0 | 2, 0 | 0, 2 | 1, 0 | 2, 0 |
| 29/6 | 0, 1 | 0, 14 | 0, 2 | 2, 0 | 0, 1 | 0, 2 |
| 30/6 | 2, 0 | 8, 0 | 2, 0 | 0, 2 | 0, 0 | 2, 0 |
| Pistillate-phase flowers available for geitonogamy | 0 | 1 | 1 | 2 | 0 | 0 |
